# Prevalence of vitamin B12 deficiency in type 2 diabetic patients taking metformin, a cross-sectional study in primary healthcare

**DOI:** 10.3389/fendo.2023.1226798

**Published:** 2023-09-04

**Authors:** Thekraiat Al Quran, Ateka Khader, Hadeel Allan, Rua’a Al-Momani, Hamzeh T. Aqel, Mu’taz Alsaleh, Ziad Bataineh

**Affiliations:** ^1^ Faculty of Medicine, Department of Public Health and Family Medicine, Jordan University of Science and Technology, Irbid, Jordan; ^2^ Hijjawi Faculty for Engineering Technology, Department of Biomedical Systems and Informatics Engineering, Yarmouk University, Irbid, Jordan; ^3^ Faculty of Medicine, Department of Anesthesia and Recovery, Jordan University of Science and Technology, Irbid, Jordan; ^4^ Faculty of Medicine, Department of General Surgery, Jordan University of Science and Technology, Irbid, Jordan; ^5^ Department of General Surgery, Dr. Sulaiman Al Habib Medical Group, Riyadh, Saudi Arabia

**Keywords:** metformin, type 2 diabetes mellitus, vitamin B 12 deficiency, Jordan, vitamin B12

## Abstract

**Background:**

Several studies documented that metformin use contributes to vitamin B12 deficiency in patients with type 2 diabetes mellitus (T2DM). However, there has been a lack of data assessing this issue in Jordan.

**Aims:**

Assess the vitamin B12 serum levels, frequency of vitamin B12 deficiency, and related factors among Jordanian patients with T2DM patients treated with metformin.

**Methods:**

a total of 447 subjects attending a primary health care center were included in this cross-sectional study consisting of T2DM patients who use metformin and a control group of non-diabetics. Serum B12 levels were evaluated and B12 deficiency was defined as serum B12 levels ≤ 200 pmol/L. Associations of B12 serum levels or B12 deficiency with other factors like gender, age, and duration of T2DM were analyzed.

**Results:**

There was no significant difference in serum B12 levels nor the frequency of vitamin B12 deficiency between T2DM metformin-treated patients and control groups. Among metformin-treated patients there was no difference relating to age, type 2 diabetes mellitus duration, proton pump inhibitors use, and metformin use (duration, dose) between patients with or without B12 deficiency.

**Conclusion:**

The prevalence of vitamin B12 deficiency among T2DM patients on metformin treatment in this study was high (48.9%). However, the treatment with metformin and the dose of metformin use was not associated with vitamin B12 deficiency.

## 1 Introduction

Biguanides (e.g. metformin) is still the first-line oral hypoglycemic agent used to manage type 2 diabetes mellitus (T2DM) (1). Gastrointestinal upset is one of the commonly reported side effects of metformin use (2); however, vitamin b12 deficiency is an underestimated one (3). The clinical manifestations of vitamin B12 deficiency can range in severity from asymptomatic or symptomatic megaloblastic anemia up to neurological complications which include peripheral neuropathy (4–6).

The association between metformin use and the decrease in serum B12 has been reported by several studies (7–13). Based on analysis of data from the National Health and Nutrition Examination Survey (NHANES), 1999–2006, a higher prevalence of B12 deficiency was present in patients with diabetes using metformin compared to those not using metformin nor to those without diabetes (7). A recent systematic review has stated that serum vitamin B12 concentrations were significantly lower in patients treated with metformin than in those who received a placebo or rosiglitazone. In addition, they found that the dose of metformin is inversely related to the serum level of vitamin B12 (8). Besides, other observational studies confirmed this relation (9–12). Although there has been a study comparing vitamin B12 levels in metformin user’s vs non-users, it was surprisingly found that patients within the cases group had a lower prevalence of vitamin B12 deficiency (13).

There was a high frequency of suboptimal serum vitamin B12 levels in an adult Jordanian (14). In addition, a population-based study reported that one-third of Jordanian adults have vitamin B12 deficiency regardless of gender (15). Vitamin B12 deficiency among Jordanians was found to be associated with low dairy intake, older ages, recurrent headaches, heartburn, peptic ulcer disease and Hpylori infection (16, 17), while vitamin B complex or multivitamins supplements would be protective (15). Based on the available evidence, the relation between vitamin B12 deficiency in T2DM patients taking metformin is not addressed in Jordan.

## 2 Method

### 2.1 Study design

A cross-sectional study was conducted involving subjects with T2DM who attended and were followed up at the Jordan University for Science & Technology (JUST) Health Center in Irbid City, between August 2021 to October 2022. All Patients with T2DM who had been treated with metformin prior to the study were screened.

Inclusion criteria: Patients aged 18 years old and above previously diagnosed with T2DM based on the ADA criteria (1), who are on metformin for at least one year and have at least one laboratory measurement of vitamin B12 during the last 3 months.

Exclusion criteria: Patients who have been diagnosed with type 1 diabetes Mellitus or LADA, pernicious anemia; prior bariatric surgery, gastrectomy, colectomy, or inflammatory bowel disease; ongoing critical illnesses; malignancy; liver cirrhosis or renal impairment will be excluded. Subjects who are recipients of vitamin B12 injections or supplements within the past 6 months, and those who is pregnant or lactating. The control group should neither have T2DM nor any other disease that may affect the Vitamin B12 level.

### 2.2 Data collection and laboratory measurements

The electronic medical records of eligible participants were reviewed. The following details were recorded per patient: age, sex, duration of T2DM, Vitamin B12 level, the dose of metformin, the duration of metformin use, and drug history (proton pump inhibitors (PPIs) or histamine H2 antagonists). Duration of T2DM and total metformin use were documented in electronic medical record\section of chronic diseases and drugs. The metformin dose had been documented during the last year.

Eligible Patients should be on metformin for at least one year. The subjects with T2DM and the control group should have measured the B12 level within the last 3 months. The vitamin B12 levels were recorded from the medical record within the last 3 months, then we went back 6 months before that date to observe if the patient took B12 supplements, Lansoprazole, or H2 blockers. Serum B12 levels were quantified using a chemiluminescent enzyme immunoassay (Access Immunoassay Systems, Beckman Coulter Inc., CA, USA). Biochemical B12 deficiency was defined as serum B12 levels ≤ 200 pmol/L which is adopted at the Jordan University for Science & Technology Health Center lab.

### 2.3 Statistical analysis

A prior power analysis, using data from a previous study by Reinstatler et al. (7),, was conducted using G*Power 3.1.9 software (Heinrich Heine University Düsseldorf, Düsseldorf, Germany), which suggested a planned sample size of n = 210 per group would be sufficient to estimate the prevalence of vitamin B12 deficiency within a margin error of 5% (α = 0.05) and 80% power. 447 subjects were included in this study, comprising 231 T2DM patients who use metformin and 216 healthy subjects. The results are presented as mean with standard deviations for continuous variables and count with proportions for categorical variables. Statistical analysis was performed using Statistical Package for the Social Science (SPSS, v.21.0, SPSS Inc, Chicago, IL). An independent t-test was used to evaluate the differences between the mean of two continuous variables. Associations between continuous variables and B12 deficiency were determined by an Independent t-test. Associations between categorical variables and B12 deficiency were determined by the Chi-square test. A one-way analysis of variance (ANOVA) test was used to determine the significance of more than two variables. The results were considered statistically significant if P < 0.05

### 2.4 Ethical considerations

Ethical approval was obtained from The Institutional Review Board (IRB) at the Jordan University for Science & Technology under number 20220560.

## 3 Results

### 3.1 Participants’ characteristics

**Figure 1:**
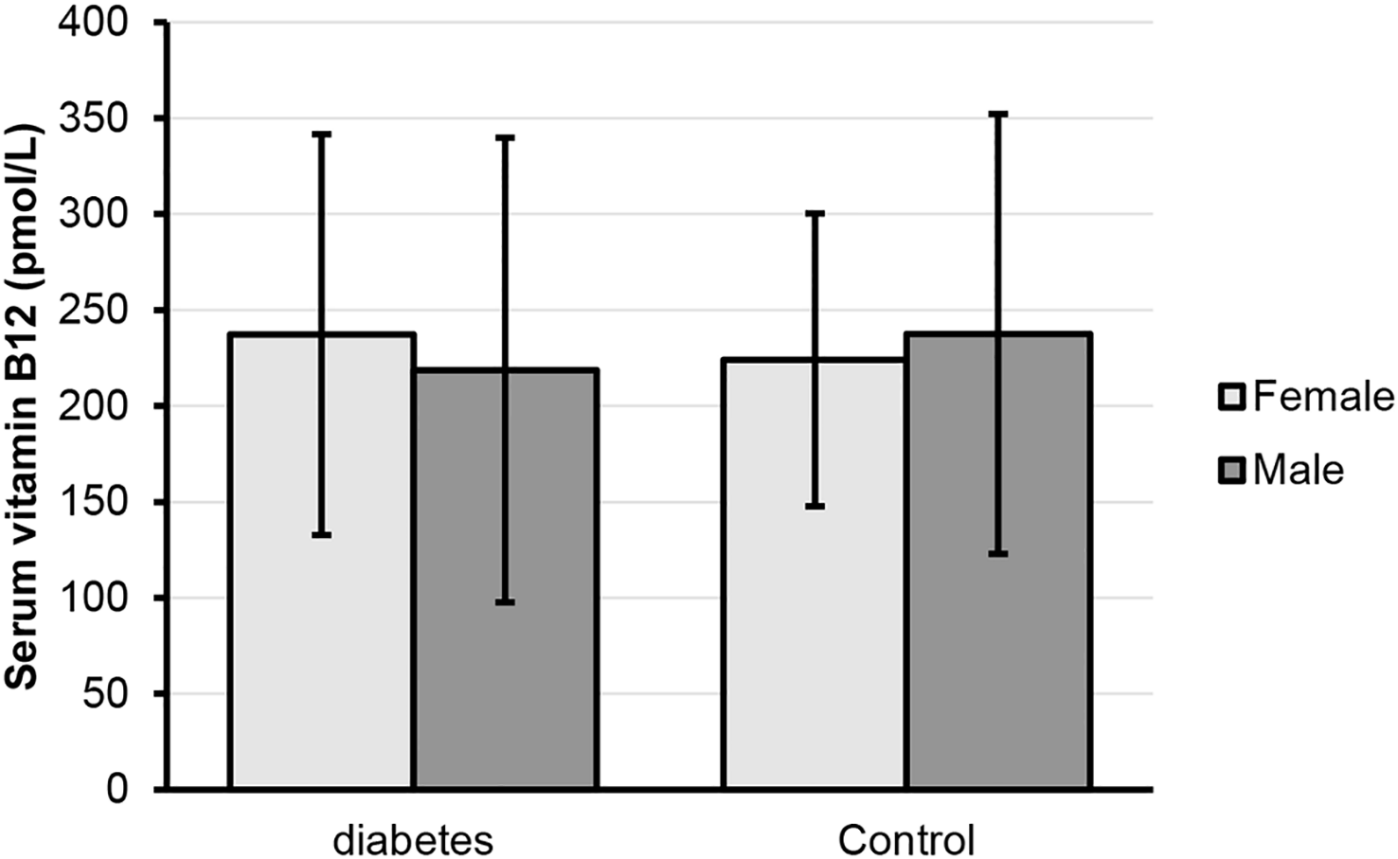
Serum vitamin B12 (pmol/L) for females and males in diabetes and control groups.

A total of 447 subjects were involved in this study, including 231 T2DM patients who use metformin and 216 without diabetes (control). Characteristics of the T2DM patients and control are shown in **Table 1**. In comparison, the control ages were significantly younger than the T2DM patients, while the percentage of males in T2DM patients was higher as compared to controls. The frequency of using PPI was significantly higher in T2DM patients. Interestingly, there was no difference in serum B12 levels between T2DM metformin-treated patients and the control group (P = 0.937). In addition, no difference in the frequency of vitamin B12 deficiency was found between the T2DM patients and controls (48.9% versus 42.4%; P = 0.108). **Figure 1** shows the serum B12 levels for males and females in T2DM patients using metformin and control. There was no significant difference in B12 levels between males and females for both groups.

**Table 1:** General characteristics of type 2 diabetes mellitus patients and control.

	Control (n = 216)	Type 2 diabetes mellitus - Metformin (n = 231)	P value
**Age (years)**	27.3 ± 9.8	59.2 ± 10.2	P < 0.0001^*^
Sex (Male, n (%))	64 (29.6%)	124 (53.7%)	P < 0.0001^**^
**Proton pump inhibitor use, n (%)**	17 (7.9%)	85 (36.8%)	P < 0.0001^**^
**Serum vitamin B12 (pmol/L)**	228.06 ± 89	227.3 ± 113.8	0.937^*^
**Vitamin B12 deficiency, n (%)**	92 (42.4%)	113 (48.9%)	0.108^**^

Data are represented as mean ± standard deviation for continuous variables and as a count (with proportions) for categorical variables. ^*^An independent t-test was done for continuous variables; ^**^ Pearson Chi-Square was done for categorical variables.

### 3.2 Type 2 diabetes mellitus patients

The characteristics of T2DM metformin-treated patients with or without B12 deficiency were further analyzed as shown in **Table 2**. There was no significant difference regarding age, T2DM duration, or metformin use duration between patients with and without B12 deficiency. Furthermore, there was no significant difference in the frequency of PPI use, or metformin use for more than 10 years among B12 deficient patients as compared to nondeficient patients. The prevalence of PPI use was more frequent in the B12-deficient group but not significant (40.7% versus 33%; P = 0.275). For the dose of metformin, 4 cases were excluded since they only took the low dose (850 mg) and we calculated the percentage of patients using 2,250 mg as compared to 1,700 mg. The prevalence of B12 deficiency among metformin-treated patients was not related to the dose of metformin (P = 0.133). The use of metformin for more than 10 years did not differ between T2DM patients with or without B12 deficiency (22.9% versus 24.8%; P = 0.753). Furthermore, the association between B12 levels and age, PPI blocker, the dose of metformin, duration of use of metformin and duration of T2DM was studied as shown in **Table 3**. Considering T2DM metformin-treated patients, there was no significant difference in B12 levels between PPI users compared to patients who did not use them (P = 0.224). Using a different dose of metformin, metformin use duration and diabetes duration also didn’t affect B12 levels.

**Table 2:** Clinical and laboratory characteristics of type 2 diabetes mellitus patients with and without vitamin B12 deficiency.

	Without vitamin B12 deficiency (n = 118)	With vitamin B12 deficiency (n = 113)	P value
**Age (years)**	59.9 ± 10.4	58.5 ± 10	0.313
**Sex (Male, n(%))**	58 (49.2%)	66 (58.4%)	0.159
**Diabetes duration (years)**	8.4 ± 5.5	7.93 ± 4.9	0.540
**Duration of metformin (years)**	6.96 ± 5.4	7.08 ± 4.6	0.856
**Proton pump inhibitor (PPI) use, n(%)**	39 (33%)	46 (40.7%)	0.275
**Dose of Metformin (2250, n(%))**	65 (56%)	73 (65.8%)	0.133
**Use of Metformin ≥ 10 years (n(%))**	28 (24.8%)	27 (22.9%)	0.753

**Table 3:** Serum B12 (pmol/L) for clinical characteristics of type 2 diabetes mellitus patients.

Characteristics		Serum B12 (pmol/L)	P Value
**PPI blocker use**			0.224
	with	234.2 ± 118.8	
	without	215.35 ± 104.8	
**Age**			0.743
	≤ 50 years	220.2 ± 103.6	
	> 50 years	226.4 ± 107.7	
**Dose of Metformin**			0.580
	1700 mg	231.6 ± 102.6	
	2550 mg	223.1 ± 118.1	
**Type 2 diabetes mellitus duration**			0.627
	< 5years	239.2 ± 113.8	
	5-10 years	222.4 ± 117	
	> 10 years	224.1 ± 106.7	
**Metformin duration**			0.393
	MET < 5years	234.7 ± 109.9	
	MET5-10 years	229.4 ± 123.9	
	MET > 10 years	204.1 ± 85.2	

PPI, proton pump inhibitors; MET, Metformin.

## 4 Discussion

In this study, the level of vitamin B12 was compared between T2DM patients on metformin and healthy non-diabetic patients at the level of primary healthcare setting. The findings of this study showed no significant difference in B12 serum levels between T2DM patients on metformin and controls. Also, there was no connection between vitamin B12 deficiency and the dose of metformin or metformin use duration or T2DM duration. To our knowledge, this study is the first one in Jordan that assessed the relationship between metformin use in T2DM patients and vitamin B12 deficiency. So, these results could be of great interest to medical care in Jordan since it seems that B12 deficiency is still prevalent among Jordanians, with or without T2DM.

The prevalence of vitamin B12 deficiency in our overall study reached almost 46%, which is higher than the last reported number (30.1%) in Jordan 2014 from a study at a national level (15), but closer to older ones (around (45%) (14, 18). This variation may be attributed to the different cut-off points and sample sizes that have been used by those reports.

Among T2DM metformin-treated patients the prevalence of vitamin B12 deficiency was 48.9%, which is relatively high in comparison to the other studies worldwide (7, 8, 12, 19–21), and other Arab countries, which ranged between (9-30%) at the level of primary and tertiary health care setting (10, 13, 22). This discrepancy in vitamin B12 deficiency rates may be related to the demographics and the dietary style of Jordanians (15, 16, 18). El-Khateeb et al. showed that the rate of vitamin B12 deficiency in the northern was higher than that in the other parts of Jordan, and our sample is from the north (15). Also Abu-Shanab et al. results revealed that Low dairy intake and some gastrointestinal diseases might be considered risk factors for having low vitamin B12 levels among Jordanians (16).

Interestingly enough, our findings showed no statistically significant difference neither in serum B12 levels nor the prevalence of vitamin B12 deficiency between T2DM metformin-treated patients and non-diabetic patients. The same outcome was detected by a study in Qatar, which reported close B12 levels between Metformin and non-Metformin users. Moreover, the prevalence of B12 deficiency was lower in the metformin-treated group than the one who was not on the drug (13). However, this is in contrast with previous studies (7, 8, 12, 19–22). A systematic review included studies that enrolled patients receiving Metformin for the treatment of T2DM or polycystic ovary syndrome, stated that serum vitamin B12 concentrations were significantly lower in patients treated with metformin than in those who received placebo or rosiglitazone (8). As well, other observational studies reported the depressed vitamin B12 level in patients on metformin treatment compared to non-metformin or non-diabetics (7, 12, 19, 22).

There were contradictory results regarding the association of the duration of metformin use or its dose among T2DM metformin-treated patients. In this study, no correlation was found between the duration of metformin use or its dose and B12 deficiency. Some researchers did not find an impact of metformin dose on vitamin B12 deficiency (12, 21), while there have been reports that documented the effect of the dose of metformin on B12 level (10, 19, 22). Cross-sectional studies found that the high daily dose of metformin > 2,000 mg could be a risk factor for metformin-associated vitamin B12 deficiency (10, 22),; moreover, another study confirmed the inverse correlation with the cumulative metformin dose and serum B12 level (19).

Reinstatler et al. assessed the prevalence of biochemical B12 deficiency in relation to the duration of metformin therapy by different intervals (< 1, > = 1-3, > 3-10,> 10 years), there was no notable increase in the prevalence of B12 deficiency as the duration of metformin use increased (7). Besides, more studies confirmed the same finding (10, 21). Though, a duration of metformin treatment of more than 4 years, or more than five years, or ≥ 10 years has been presented as an associated factor for B12 deficiency by other reports, respectively (11, 12, 22). This controversy may be due to the variation in patient characteristics, as it is notable that the percent of our cases who used Metformin ≥ 10 years or took the high dose of Metformin (2,250 mg) was relatively small (15.6% and 56.4% respectively).

For B12 deficient and non-deficient T2DM patients, there were no differences related to age and the duration of T2DM. Previous studies arrived at the same finding (10, 12), Nonetheless, a prospective study reported younger age and short duration of diabetes were significantly correlated with B12 deficiency (13). The association of using PPI drugs with vitamin B12 deficiency was investigated by former studies, which was inconclusive. In this study, no linkage was found between using PPI drugs and vitamin B12 deficiency in T2DM metformin-treated patients. Calvo Romero et al. and Ko et al. found also no negative effect of using PPI drugs on vitamin B12 deficiency (21, 23). While PPI was recognized as a risk factor for metformin-associated vitamin B12 deficiency by some reports (10, 12), it’s use reduced the odds of vitamin B12 deficiency by other researchers (24).

The prevalence of vitamin B12 deficiency among T2DM metformin-treated patients in Jordan is higher than that in other reported studies. Interestingly, there was no association between developing B12 deficiency and using metformin in T2DM patients. Diet factors of Jordanians and precise cutoff point for diagnosing B12 deficiency are advised to be investigated more at the national level. However, this study has some limitations. It included a single primary healthcare center which may not be representative of the whole Jordanian population. In addition, the significantly younger age of controls and the imbalance of the gender distribution between cases and controls were considered limitation factors. It was a cross-sectional study as it depended on patients’ electronic medical records. Moreover, other risk factors for vitamin B12 deficiency such as the details of the patient’s diet, and history of over-the-counter vitamin supplements were not included. Therefore, larger prospective studies are needed to address a larger sample to focus more on dietary habits.

## Data availability statement

The raw data supporting the conclusions of this article will be made available by the authors, without undue reservation.

## Ethics statement

The studies involving humans were approved by The Institutional Review Board (IRB) at the Jordan University for Science & Technology. The studies were conducted in accordance with the local legislation and institutional requirements. The ethics committee/institutional review board waived the requirement of written informed consent for participation from the participants or the participants’ legal guardians/next of kin because of the retrospective design of the study.

## Author contributions

Conceptualization, TQ, ZB; methodology, TQ, and AK; software, and formal analysis;AK, investigation and resources, HA, MA, HTA, and RA-M; data curation, HTA, RA-M and MA; writing—original draft preparation, TQ, AK, RA-M, HA, and ZB; writing—review and editing, TA, ZB, and AK; supervision, TA, RA-M, and HA. All authors read and approved the final submitted and revised manuscript.

## References

[1] American Diabetes Association. Standards of medical care in diabetes – 2016 abridged for primary care providers. Clin Diabetes (2016) 34(1):3–21. doi: 10.2337/diaclin.34.1.3 26807004 PMC4714725

[2] Bonnet F Scheen A. Understanding and overcoming metformin gastrointestinal intolerance. Diabetes Obes Metab (2017) 19(4):473–81. doi: 10.1111/dom.12854 27987248

[3] Ahmed MA. Metformin and vitamin B12 deficiency: where do we stand? J Pharm Pharm Sci (2016) 19(3):382–98. doi: 10.18433/j3pk7p 27806244

[4] Lindenbaum J Healton EB Savage DG Brust JC Garrett TJ Podell ER. Neuropsychiatric disorders caused by cobalamin deficiency in the absence of anemia or macrocytosis. N Engl J Med (1988) 318(26):1720–8. doi: 10.1056/nejm198806303182604 3374544

[5] Devalia V Hamilton MS Molloy AM British Committee for Standards in Haematology. Guidelines for the diagnosis and treatment of cobalamin and folate disorders. Br J Haematol (2014) 166(4):496–513. doi: 10.1111/bjh.12959 24942828

[6] Stein J Geisel J Obeid R. Association between neuropathy and B-vitamins: A systematic review and meta-analysis. Eur J Neurol (2021) 28(6):2054–64. doi: 10.1111/ene.14786 33619867

[7] Reinstatler L Qi YP Williamson RS Garn JV Oakley GP Jr. Association of biochemical B_12_ deficiency with metformin therapy and vitamin B_12_ supplements: the National Health and Nutrition Examination Survey, 1999-2006. Diabetes Care (2012) 35(2):327–33. doi: 10.2337/dc11-1582 PMC3263877 22179958

[8] Liu Q Li S Quan H Li J. Vitamin B12 status in metformin treated patients: systematic review. PloS One (2014) 9(6):e100379. doi: 10.1371/journal.pone.0100379 24959880 PMC4069007

[9] Kos E Liszek MJ Emanuele MA Durazo-Arvizu R Camacho P. Effect of metformin therapy on vitamin D and vitamin B_12_ levels in patients with type 2 diabetes mellitus. Endocr Pract (2012) 18(2):179–84. doi: 10.4158/ep11009.or 21940283

[10] Yousef Khan F Yousif AB Suliman A Saleh AO Magdi M Alshurafa A. Association of vitamin B12 deficiency with metformin use in patients with type 2 diabetes treated in the largest tertiary care hospital in Qatar. Qatar Med J (2021) 2021(2):39. doi: 10.5339/qmj.2021.39 34540601 PMC8428509

[11] Krishnan GD Zakaria MH Yahaya N. Prevalence of Vitamin B12 Deficiency and its Associated Factors among Patients with Type 2 Diabetes Mellitus on Metformin from a District in Malaysia. J ASEAN Fed Endocr Soc (2020) 35(2):163–8. doi: 10.15605/jafes.035.02.03 PMC7784158 33442187

[12] Damião CP Rodrigues AO Pinheiro MFMC Cruz Filho RA Cardoso GP Taboada GF. Prevalence of vitamin B12 deficiency in type 2 diabetic patients using metformin: a cross-sectional study. Sao Paulo Med J (2016) 134(6):473–9. doi: 10.1590/1516-3180.2015.01382111 PMC11448733 28076635

[13] Elhadd T Ponirakis G Dabbous Z Siddique M Chinnaiyan S Malik RA. Metformin use is not associated with B12 deficiency or neuropathy in patients with type 2 diabetes mellitus in Qatar. Front Endocrinol (2018) 9:248. doi: 10.3389/fendo.2018.00248 PMC5980977 29887831

[14] Fora MA Mohammad MA. High frequency of suboptimal serum vitamin B12 level in adults in Jordan. Saudi Med J (2005) 26(10):1591–5. 16228062

[15] El-Khateeb M Khader Y Batieha A Jaddou H Hyassat D Belbisi A. Vitamin B12 deficiency in Jordan: a population-based study. Ann Nutr Metab (2014) 64(2):101–5. doi: 10.1159/000355440 24943588

[16] Abu-Shanab A Zihlif M Rbeihat MN Shkoukani ZW Khamis A Isleem U. Vitamin B12 deficiency among the healthy Jordanian adult population: diagnostic levels, symptomology and risk factors. Endocr Metab Immune Disord Drug Targets. (2021) 21(6):1107–14. doi: 10.2174/1871530320999200831230205 32875992

[17] Ayesh MH Jadalah K Al Awadi E Alawneh K Khassawneh B. Association between vitamin B12 level and anti-parietal cells and anti-intrinsic factor antibodies among adult Jordanian patients with Helicobacter pylori infection. Braz J Infect Dis (2013) 17(6):629–32. doi: 10.1016/j.bjid.2013.01.009 PMC9427375 23746879

[18] Barghouti FF Younes NA Halaseh LJ Said TT Ghraiz SM. High frequency of low serum levels of vitamin 12 among patients attending Jordan University Hospital. East Mediterr Health J (2009) 15(4):853–60. doi: 10.26719/2009.15.4.853 20187536

[19] Wile DJ Toth C. Association of metformin, elevated homocysteine, and methylmalonic acid levels and clinically worsened diabetic peripheral neuropathy. Diabetes Care (2010) 33(1):156–61. doi: 10.2337/dc09-0606 PMC2797962 19846797

[20] Pflipsen MC Oh RC Saguil A Seehusen DA Seaquist D Topolski R. The prevalence of vitamin B(12) deficiency in patients with type 2 diabetes: a cross-sectional study. J Am Board Fam Med (2009) 22(5):528–34. doi: 10.3122/jabfm.2009.05.090044 19734399

[21] Calvo Romero JM Ramiro Lozano JM. Vitamin B(12) in type 2 diabetic patients treated with metformin. Endocrinol Nutr (2012) 59(8):487–90. doi: 10.1016/j.endonu.2012.06.005 22981397

[22] Alharbi TJ Tourkmani AM Abdelhay O Alkhashan HI Al-Asmari AK Bin Rsheed AM. The association of metformin use with vitamin B12 deficiency and peripheral neuropathy in Saudi individuals with type 2 diabetes mellitus. PloS One (2018) 13(10):e0204420. doi: 10.1371/journal.pone.0204420 30321183 PMC6188756

[23] Ko SH Ko SH Ahn YB Song KH Han KD Park YM. Association of vitamin B12 deficiency and metformin use in patients with type 2 diabetes. J Korean Med Sci (2014) 29(7):965–72. doi: 10.3346/jkms.2014.29.7.965 PMC4101785 25045229

[24] de Groot-Kamphuis DM van Dijk PR Groenier KH Houweling ST Bilo HJ Kleefstra N. Vitamin B12 deficiency and the lack of its consequences in type 2 diabetes patients using metformin. Neth J Med (2013) 71(7):386–90. 24038568

